# Methyl 6-(4-chloro­phen­yl)-2,4-dimethyl-1,3-dioxo-1,2,3,4,6,6a,7,12b-octa­hydro­chromeno[4′,3′:4,5]pyrano[2,3-*d*]pyrimidine-6a-carboxyl­ate

**DOI:** 10.1107/S160053681102678X

**Published:** 2011-07-09

**Authors:** J. Kanchanadevi, G. Anbalagan, G. Sivakumar, M. Bakthadoss, V. Manivannan

**Affiliations:** a Department of Physics, Velammal Institute of Technology, Panchetty, Chennai 601 204, India; b Department of Physics, Presidency College (Autonomous), Chennai 600 005, India; c Department of Organic Chemistry, University of Madras, Maraimalai campus, Chennai 600 025, India; d Department of Research and Development, PRIST University, Vallam, Thanjavur 613 403, Tamil Nadu, India

## Abstract

In the title compound, C_24_H_21_ClN_2_O_6_, the two fused six-membered pyran rings adopt half-chair conformations. The dihedral angle between the pyrimidine ring and the chloro­phenyl ring is 51.55 (3)°. In the crystal, mol­ecules are linked by pairs of weak inter­molecular C—H⋯O hydrogen bonds, forming inversion dimers. A C—H⋯π inter­action is also observed.

## Related literature

For biological activity of pyrimidine derivatives, see: Alam *et al.* (2005 ▶); Kappe (2000 ▶); Condon *et al.* (1993 ▶); Rovnyak *et al.* (1995 ▶); Leite *et al.* (2006 ▶); Sriram *et al.* (2006 ▶). For related structures, see: Booysen *et al.* (2011 ▶); Noroozi Pesyan *et al.* (2009 ▶).
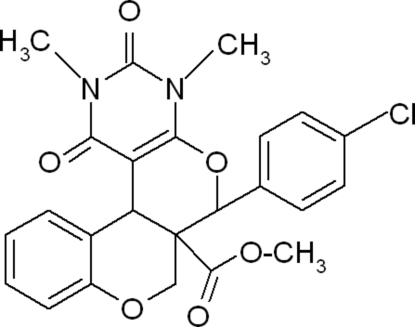


## Experimental

### 

#### Crystal data

C_24_H_21_ClN_2_O_6_
*M* *_r_* = 468.88
Monoclinic, 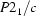

*a* = 10.6177 (5) Å
*b* = 11.9973 (5) Å
*c* = 17.5532 (8) Å
β = 99.751 (2)°
*V* = 2203.69 (17) Å^3^
*Z* = 4
Mo *K*α radiation
μ = 0.22 mm^−1^
*T* = 295 K
0.30 × 0.25 × 0.20 mm

#### Data collection

Bruker Kappa APEXII CCD diffractometer
Absorption correction: multi-scan (*SADABS*; Sheldrick, 1996 ▶) *T*_min_ = 0.924, *T*_max_ = 0.951
29343 measured reflections
7232 independent reflections
4518 reflections with *I* > 2σ(*I*)
*R*_int_ = 0.029

#### Refinement

*R*[*F* ^2^ > 2σ(*F* ^2^)] = 0.052
*wR*(*F* ^2^) = 0.158
*S* = 1.03
7232 reflections
301 parameters
H-atom parameters constrained
Δρ_max_ = 0.42 e Å^−3^
Δρ_min_ = −0.60 e Å^−3^

### 

Data collection: *APEX2* (Bruker, 2004 ▶); cell refinement: *SAINT* (Bruker, 2004 ▶); data reduction: *SAINT*; program(s) used to solve structure: *SHELXS97* (Sheldrick, 2008 ▶); program(s) used to refine structure: *SHELXL97* (Sheldrick, 2008 ▶); molecular graphics: *PLATON* (Spek, 2009 ▶); software used to prepare material for publication: *SHELXL97*.

## Supplementary Material

Crystal structure: contains datablock(s) global, I. DOI: 10.1107/S160053681102678X/is2742sup1.cif

Structure factors: contains datablock(s) I. DOI: 10.1107/S160053681102678X/is2742Isup2.hkl

Supplementary material file. DOI: 10.1107/S160053681102678X/is2742Isup3.cml

Additional supplementary materials:  crystallographic information; 3D view; checkCIF report

## Figures and Tables

**Table 1:** Hydrogen-bond geometry (Å, °) *Cg*4 is the centroid of the C1–C5/C9 ring.

*D*—H⋯*A*	*D*—H	H⋯*A*	*D*⋯*A*	*D*—H⋯*A*
C16—H16⋯O3^i^	0.93	2.44	3.349 (2)	166
C19—H19⋯*Cg*4^ii^	0.93	2.84	3.720 (3)	158

Symmetry codes: (i) 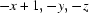; (ii) 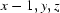.

**Fig. 1.**
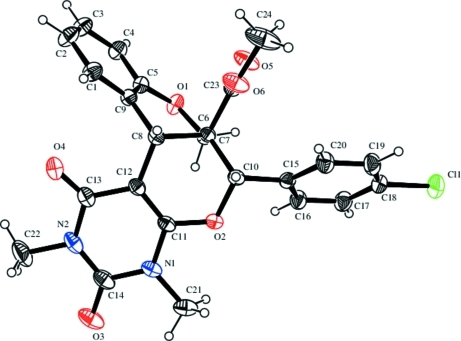
The molecular structure of the title compound, with atom labels and 30% probability displacement ellipsoids for non-H atoms.

**Fig. 2.**
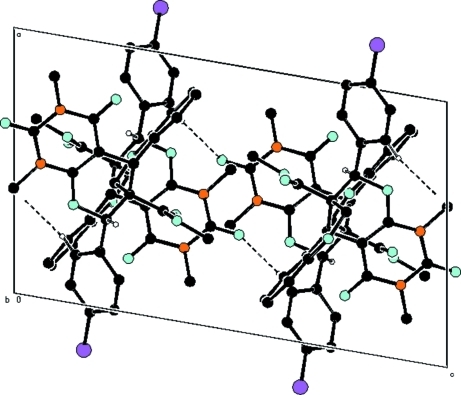
A packing diagram of the title compound, viewed down the b axis. Hydrogen bonds are shown as dashed lines.

## supplementary crystallographic information

### Comment

Pyrimidine derivatives are used in the areas of pesticide and pharmaceutical agents (Condon *et al.*, 1993). In addition, pyrimidine-2(1*H*)-ones/thiones are calcium channel blocker compounds (Rovnyak *et al.*, 1995). They also have other biological activities such as antibacterial, antifungal and antiviral (Kappe, 2000; Alam *et al.*, 2005; Sriram *et al.*, 2006; Leite *et al.*, 2006).

The geometric parameters of the title molecule (Fig. 1) agree well with reported similar structure (Booysen *et al.*, 2011; Noroozi Pesyan *et al.*, 2009). The sum of bond angles around N1 and N2 [359.93 (15) and 358.92°, respectively] indicates the *sp^2^* hybridization state of atoms N1 and N2 in the molecule. The crystal packing is controlled by weak intermolecular C—H···O and C—H···π interactions (Table 1).

### Experimental

A mixture of (*E*)-methyl 2-((2-formylphenoxy)methyl)-3-(4-chlorophenyl)acrylate (0.330 g, 1 mmol) and *N*,*N*-dimethylbarbutric acid (0.156 g, 1 mmol) was placed in a round bottom flask and melted at 180 °C for 1 h. After completion of the reaction as indicated by TLC, the crude product was washed with 5 ml of ethylacetate and hexane mixture (1:49 ratio) which successfully provided the pure product methyl-6-(4-chlorophenyl)-2,4-dimethyl-1,3-dioxo- 1,2,3,4,6,6a,7,12octahydrochromeno[4',3',4,5]pyrano[2,3-*d*]pyrimidine- 6a-carboxylate, as colorless solid in 96% yield.

### Refinement

H atoms were positioned geometrically and refined using riding model with C—H = 0.93 Å and *U*_iso_(H) = 1.2*U*_eq_(C) for aromatic CH, C—H = 0.98 Å and *U*_iso_(H) = 1.2*U*_eq_(C) for methine CH, C—H = 0.97 Å and *U*_iso_(H) = 1.2*U*_eq_(C) for CH_2_, C—H = 0.96 Å and *U*_iso_(H) = 1.5*U*_eq_(C) for CH_3_.

### Crystal data

**Table:**

C_24_H_21_ClN_2_O_6_	*F*(000) = 976
*M**_r_* = 468.88	*D*_x_ = 1.413 Mg m^−^^3^
Monoclinic, *P*2_1_/*c*	Mo *K*α radiation, λ = 0.71073 Å
Hall symbol: -P 2ybc	Cell parameters from 7610 reflections
*a* = 10.6177 (5) Å	θ = 2.2–30.2°
*b* = 11.9973 (5) Å	µ = 0.22 mm^−^^1^
*c* = 17.5532 (8) Å	*T* = 295 K
β = 99.751 (2)°	Block, colourless
*V* = 2203.69 (17) Å^3^	0.30 × 0.25 × 0.20 mm
*Z* = 4	

### Data collection

**Table:**

Bruker Kappa APEXII CCD diffractometer	7232 independent reflections
Radiation source: fine-focus sealed tube	4518 reflections with *I* > 2σ(*I*)
graphite	*R*_int_ = 0.029
Detector resolution: 0 pixels mm^-1^	θ_max_ = 31.7°, θ_min_ = 2.1°
ω and φ scans	*h* = −15→15
Absorption correction: multi-scan (*SADABS*; Sheldrick, 1996)	*k* = −15→17
*T*_min_ = 0.924, *T*_max_ = 0.951	*l* = −25→25
29343 measured reflections	

### Refinement

**Table:**

Refinement on *F*^2^	Primary atom site location: structure-invariant direct methods
Least-squares matrix: full	Secondary atom site location: difference Fourier map
*R*[*F*^2^ > 2σ(*F*^2^)] = 0.052	Hydrogen site location: inferred from neighbouring sites
*wR*(*F*^2^) = 0.158	H-atom parameters constrained
*S* = 1.03	*w* = 1/[σ^2^(*F*_o_^2^) + (0.0706*P*)^2^ + 0.6194*P*] where *P* = (*F*_o_^2^ + 2*F*_c_^2^)/3
7232 reflections	(Δ/σ)_max_ = 0.005
301 parameters	Δρ_max_ = 0.42 e Å^−^^3^
0 restraints	Δρ_min_ = −0.60 e Å^−^^3^

### Fractional atomic coordinates and isotropic or equivalent isotropic displacement parameters (Å^2^)

**Table:**

	*x*	*y*	*z*	*U*_iso_*/*U*_eq_	
C1	0.76555 (18)	0.06202 (16)	0.37637 (10)	0.0480 (4)	
H1	0.7599	0.1389	0.3819	0.058*	
C2	0.8645 (2)	0.00508 (18)	0.42011 (11)	0.0568 (5)	
H2	0.9268	0.0436	0.4536	0.068*	
C3	0.87131 (19)	−0.10942 (17)	0.41423 (11)	0.0541 (5)	
H3	0.9383	−0.1480	0.4438	0.065*	
C4	0.77967 (17)	−0.16628 (15)	0.36499 (10)	0.0459 (4)	
H4	0.7837	−0.2435	0.3616	0.055*	
C5	0.68080 (14)	−0.10821 (13)	0.32016 (9)	0.0361 (3)	
C6	0.47962 (14)	−0.12010 (12)	0.23307 (8)	0.0338 (3)	
H6A	0.4081	−0.1706	0.2327	0.041*	
H6B	0.4906	−0.1088	0.1799	0.041*	
C7	0.44663 (14)	−0.00850 (12)	0.26651 (8)	0.0310 (3)	
C8	0.56484 (14)	0.06889 (12)	0.27607 (8)	0.0328 (3)	
H8	0.5464	0.1355	0.3046	0.039*	
C9	0.67411 (15)	0.00706 (13)	0.32418 (9)	0.0351 (3)	
C10	0.33697 (14)	0.05058 (13)	0.21285 (8)	0.0338 (3)	
H10	0.3240	0.1238	0.2350	0.041*	
C11	0.49137 (15)	0.10189 (13)	0.13570 (9)	0.0362 (3)	
C12	0.58675 (15)	0.10449 (13)	0.19648 (9)	0.0356 (3)	
C13	0.70722 (17)	0.15057 (14)	0.18445 (11)	0.0438 (4)	
C14	0.6217 (2)	0.16628 (16)	0.04577 (11)	0.0510 (4)	
C15	0.21085 (14)	−0.00924 (13)	0.19859 (9)	0.0356 (3)	
C16	0.18358 (16)	−0.09052 (16)	0.14232 (10)	0.0464 (4)	
H16	0.2442	−0.1090	0.1119	0.056*	
C17	0.06716 (18)	−0.14441 (18)	0.13101 (11)	0.0529 (5)	
H17	0.0493	−0.1992	0.0932	0.063*	
C18	−0.02226 (16)	−0.11680 (17)	0.17584 (11)	0.0482 (4)	
C19	0.00161 (17)	−0.03659 (19)	0.23166 (12)	0.0550 (5)	
H19	−0.0595	−0.0188	0.2619	0.066*	
C20	0.11761 (17)	0.01751 (16)	0.24245 (11)	0.0483 (4)	
H20	0.1339	0.0730	0.2798	0.058*	
C21	0.3952 (2)	0.13297 (19)	−0.00072 (10)	0.0597 (5)	
H21A	0.3603	0.0592	−0.0081	0.090*	
H21B	0.4218	0.1577	−0.0475	0.090*	
H21C	0.3312	0.1828	0.0123	0.090*	
C22	0.8425 (2)	0.2140 (3)	0.09356 (16)	0.0834 (8)	
H22A	0.8659	0.1797	0.0486	0.125*	
H22B	0.9071	0.1990	0.1376	0.125*	
H22C	0.8348	0.2931	0.0857	0.125*	
C23	0.40621 (15)	−0.02757 (14)	0.34447 (9)	0.0364 (3)	
C24	0.3303 (3)	0.0581 (2)	0.44840 (13)	0.0941 (10)	
H24A	0.3101	−0.0184	0.4569	0.141*	
H24B	0.2547	0.1028	0.4461	0.141*	
H24C	0.3943	0.0835	0.4901	0.141*	
N1	0.50522 (15)	0.13185 (13)	0.06206 (8)	0.0447 (3)	
N2	0.71979 (16)	0.16862 (13)	0.10700 (10)	0.0523 (4)	
O1	0.59218 (11)	−0.17168 (9)	0.27413 (7)	0.0444 (3)	
O2	0.37171 (11)	0.06736 (10)	0.13744 (6)	0.0408 (3)	
O3	0.63502 (17)	0.19131 (14)	−0.01960 (8)	0.0738 (5)	
O4	0.79552 (13)	0.17313 (13)	0.23567 (9)	0.0621 (4)	
O5	0.39933 (16)	−0.11577 (11)	0.37358 (8)	0.0623 (4)	
O6	0.37805 (15)	0.06763 (11)	0.37639 (7)	0.0581 (4)	
Cl1	−0.16887 (5)	−0.18537 (6)	0.16129 (4)	0.0765 (2)	

### Atomic displacement parameters (Å^2^)

**Table:**

	*U*^11^	*U*^22^	*U*^33^	*U*^12^	*U*^13^	*U*^23^
C1	0.0536 (10)	0.0389 (9)	0.0480 (9)	−0.0044 (8)	−0.0018 (8)	−0.0048 (7)
C2	0.0545 (11)	0.0559 (12)	0.0523 (10)	−0.0036 (9)	−0.0128 (8)	−0.0078 (9)
C3	0.0480 (10)	0.0566 (12)	0.0523 (10)	0.0083 (9)	−0.0069 (8)	−0.0013 (9)
C4	0.0447 (9)	0.0387 (9)	0.0527 (10)	0.0080 (7)	0.0033 (7)	−0.0013 (7)
C5	0.0350 (7)	0.0340 (8)	0.0392 (7)	0.0002 (6)	0.0056 (6)	−0.0032 (6)
C6	0.0363 (7)	0.0289 (7)	0.0357 (7)	0.0003 (6)	0.0046 (6)	−0.0008 (6)
C7	0.0342 (7)	0.0277 (7)	0.0316 (6)	0.0001 (5)	0.0076 (5)	0.0014 (5)
C8	0.0373 (7)	0.0258 (7)	0.0356 (7)	−0.0010 (6)	0.0074 (6)	−0.0005 (5)
C9	0.0371 (8)	0.0309 (8)	0.0369 (7)	−0.0015 (6)	0.0050 (6)	−0.0003 (6)
C10	0.0382 (7)	0.0314 (8)	0.0327 (7)	0.0036 (6)	0.0089 (6)	0.0025 (6)
C11	0.0462 (8)	0.0288 (8)	0.0361 (7)	0.0018 (6)	0.0142 (6)	0.0033 (6)
C12	0.0397 (8)	0.0287 (7)	0.0407 (8)	0.0012 (6)	0.0135 (6)	0.0025 (6)
C13	0.0435 (9)	0.0366 (9)	0.0549 (10)	0.0011 (7)	0.0191 (8)	0.0027 (7)
C14	0.0686 (12)	0.0424 (10)	0.0490 (10)	0.0040 (8)	0.0307 (9)	0.0009 (8)
C15	0.0343 (7)	0.0363 (8)	0.0360 (7)	0.0054 (6)	0.0057 (6)	0.0018 (6)
C16	0.0396 (8)	0.0580 (11)	0.0432 (9)	−0.0004 (8)	0.0113 (7)	−0.0102 (8)
C17	0.0461 (10)	0.0610 (12)	0.0499 (10)	−0.0055 (8)	0.0037 (8)	−0.0112 (9)
C18	0.0334 (8)	0.0594 (12)	0.0504 (9)	−0.0006 (7)	0.0030 (7)	0.0066 (8)
C19	0.0386 (9)	0.0707 (13)	0.0592 (11)	0.0044 (9)	0.0179 (8)	−0.0048 (10)
C20	0.0430 (9)	0.0516 (11)	0.0526 (10)	0.0039 (8)	0.0149 (8)	−0.0100 (8)
C21	0.0799 (14)	0.0630 (13)	0.0364 (9)	0.0073 (11)	0.0098 (9)	0.0083 (8)
C22	0.0676 (15)	0.099 (2)	0.0960 (18)	−0.0118 (13)	0.0491 (14)	0.0139 (15)
C23	0.0387 (8)	0.0362 (8)	0.0351 (7)	0.0014 (6)	0.0086 (6)	0.0029 (6)
C24	0.159 (3)	0.0829 (18)	0.0556 (13)	0.0290 (18)	0.0609 (16)	0.0027 (12)
N1	0.0588 (9)	0.0422 (8)	0.0361 (7)	0.0027 (7)	0.0168 (6)	0.0048 (6)
N2	0.0549 (9)	0.0483 (9)	0.0621 (10)	−0.0012 (7)	0.0342 (8)	0.0029 (7)
O1	0.0370 (6)	0.0292 (6)	0.0629 (7)	0.0029 (4)	−0.0030 (5)	−0.0083 (5)
O2	0.0430 (6)	0.0459 (7)	0.0333 (5)	−0.0026 (5)	0.0057 (4)	0.0076 (5)
O3	0.1000 (12)	0.0798 (11)	0.0528 (8)	0.0023 (9)	0.0455 (8)	0.0096 (7)
O4	0.0462 (7)	0.0716 (10)	0.0697 (9)	−0.0159 (7)	0.0130 (7)	0.0056 (7)
O5	0.0981 (11)	0.0404 (8)	0.0562 (8)	0.0018 (7)	0.0359 (8)	0.0126 (6)
O6	0.0926 (11)	0.0442 (7)	0.0451 (7)	0.0092 (7)	0.0330 (7)	−0.0009 (5)
Cl1	0.0427 (3)	0.1053 (5)	0.0794 (4)	−0.0208 (3)	0.0046 (2)	0.0005 (3)

### Geometric parameters (Å, °)

**Table:**

C1—C2	1.374 (3)	C13—N2	1.405 (2)
C1—C9	1.384 (2)	C14—O3	1.217 (2)
C1—H1	0.9300	C14—N2	1.364 (3)
C2—C3	1.380 (3)	C14—N1	1.379 (2)
C2—H2	0.9300	C15—C16	1.383 (2)
C3—C4	1.369 (3)	C15—C20	1.391 (2)
C3—H3	0.9300	C16—C17	1.379 (3)
C4—C5	1.388 (2)	C16—H16	0.9300
C4—H4	0.9300	C17—C18	1.372 (3)
C5—O1	1.3639 (18)	C17—H17	0.9300
C5—C9	1.387 (2)	C18—C19	1.366 (3)
C6—O1	1.4280 (18)	C18—Cl1	1.7408 (18)
C6—C7	1.526 (2)	C19—C20	1.377 (3)
C6—H6A	0.9700	C19—H19	0.9300
C6—H6B	0.9700	C20—H20	0.9300
C7—C23	1.519 (2)	C21—N1	1.464 (2)
C7—C10	1.541 (2)	C21—H21A	0.9600
C7—C8	1.547 (2)	C21—H21B	0.9600
C8—C9	1.509 (2)	C21—H21C	0.9600
C8—C12	1.516 (2)	C22—N2	1.468 (2)
C8—H8	0.9800	C22—H22A	0.9600
C10—O2	1.4471 (17)	C22—H22B	0.9600
C10—C15	1.502 (2)	C22—H22C	0.9600
C10—H10	0.9800	C23—O5	1.183 (2)
C11—C12	1.341 (2)	C23—O6	1.328 (2)
C11—O2	1.3416 (19)	C24—O6	1.444 (2)
C11—N1	1.3734 (19)	C24—H24A	0.9600
C12—C13	1.442 (2)	C24—H24B	0.9600
C13—O4	1.214 (2)	C24—H24C	0.9600
			
C2—C1—C9	121.17 (17)	O3—C14—N2	122.88 (19)
C2—C1—H1	119.4	O3—C14—N1	121.4 (2)
C9—C1—H1	119.4	N2—C14—N1	115.75 (15)
C1—C2—C3	119.86 (17)	C16—C15—C20	118.39 (16)
C1—C2—H2	120.1	C16—C15—C10	121.91 (14)
C3—C2—H2	120.1	C20—C15—C10	119.70 (15)
C4—C3—C2	120.16 (17)	C17—C16—C15	120.49 (16)
C4—C3—H3	119.9	C17—C16—H16	119.8
C2—C3—H3	119.9	C15—C16—H16	119.8
C3—C4—C5	119.75 (17)	C18—C17—C16	119.64 (18)
C3—C4—H4	120.1	C18—C17—H17	120.2
C5—C4—H4	120.1	C16—C17—H17	120.2
O1—C5—C9	123.41 (14)	C19—C18—C17	121.26 (17)
O1—C5—C4	115.80 (15)	C19—C18—Cl1	119.39 (14)
C9—C5—C4	120.79 (15)	C17—C18—Cl1	119.35 (15)
O1—C6—C7	114.40 (12)	C18—C19—C20	118.93 (17)
O1—C6—H6A	108.7	C18—C19—H19	120.5
C7—C6—H6A	108.7	C20—C19—H19	120.5
O1—C6—H6B	108.7	C19—C20—C15	121.28 (17)
C7—C6—H6B	108.7	C19—C20—H20	119.4
H6A—C6—H6B	107.6	C15—C20—H20	119.4
C23—C7—C6	109.42 (12)	N1—C21—H21A	109.5
C23—C7—C10	108.69 (12)	N1—C21—H21B	109.5
C6—C7—C10	111.56 (12)	H21A—C21—H21B	109.5
C23—C7—C8	109.90 (12)	N1—C21—H21C	109.5
C6—C7—C8	109.47 (12)	H21A—C21—H21C	109.5
C10—C7—C8	107.78 (11)	H21B—C21—H21C	109.5
C9—C8—C12	115.51 (12)	N2—C22—H22A	109.5
C9—C8—C7	107.36 (12)	N2—C22—H22B	109.5
C12—C8—C7	108.51 (12)	H22A—C22—H22B	109.5
C9—C8—H8	108.4	N2—C22—H22C	109.5
C12—C8—H8	108.4	H22A—C22—H22C	109.5
C7—C8—H8	108.4	H22B—C22—H22C	109.5
C1—C9—C5	118.19 (15)	O5—C23—O6	123.51 (15)
C1—C9—C8	121.53 (14)	O5—C23—C7	124.85 (15)
C5—C9—C8	120.18 (13)	O6—C23—C7	111.63 (13)
O2—C10—C15	105.79 (12)	O6—C24—H24A	109.5
O2—C10—C7	109.87 (11)	O6—C24—H24B	109.5
C15—C10—C7	116.24 (12)	H24A—C24—H24B	109.5
O2—C10—H10	108.2	O6—C24—H24C	109.5
C15—C10—H10	108.2	H24A—C24—H24C	109.5
C7—C10—H10	108.2	H24B—C24—H24C	109.5
C12—C11—O2	125.41 (13)	C11—N1—C14	121.08 (15)
C12—C11—N1	123.77 (15)	C11—N1—C21	120.78 (15)
O2—C11—N1	110.80 (14)	C14—N1—C21	118.08 (15)
C11—C12—C13	117.48 (14)	C14—N2—C13	125.05 (15)
C11—C12—C8	120.89 (13)	C14—N2—C22	117.44 (17)
C13—C12—C8	121.44 (14)	C13—N2—C22	116.43 (18)
O4—C13—N2	119.60 (16)	C5—O1—C6	119.44 (12)
O4—C13—C12	124.74 (16)	C11—O2—C10	116.93 (12)
N2—C13—C12	115.66 (16)	C23—O6—C24	116.01 (16)
			
C9—C1—C2—C3	−2.2 (3)	O2—C10—C15—C20	−141.61 (15)
C1—C2—C3—C4	0.0 (3)	C7—C10—C15—C20	96.17 (18)
C2—C3—C4—C5	0.9 (3)	C20—C15—C16—C17	−0.8 (3)
C3—C4—C5—O1	−178.52 (17)	C10—C15—C16—C17	179.57 (16)
C3—C4—C5—C9	0.5 (3)	C15—C16—C17—C18	0.2 (3)
O1—C6—C7—C23	−68.32 (16)	C16—C17—C18—C19	0.1 (3)
O1—C6—C7—C10	171.38 (12)	C16—C17—C18—Cl1	−179.90 (16)
O1—C6—C7—C8	52.18 (16)	C17—C18—C19—C20	0.3 (3)
C23—C7—C8—C9	65.52 (15)	Cl1—C18—C19—C20	−179.69 (16)
C6—C7—C8—C9	−54.69 (15)	C18—C19—C20—C15	−1.0 (3)
C10—C7—C8—C9	−176.20 (11)	C16—C15—C20—C19	1.3 (3)
C23—C7—C8—C12	−169.00 (12)	C10—C15—C20—C19	−179.14 (17)
C6—C7—C8—C12	70.80 (14)	C6—C7—C23—O5	−1.3 (2)
C10—C7—C8—C12	−50.71 (15)	C10—C7—C23—O5	120.72 (18)
C2—C1—C9—C5	3.5 (3)	C8—C7—C23—O5	−121.56 (18)
C2—C1—C9—C8	179.82 (17)	C6—C7—C23—O6	179.19 (14)
O1—C5—C9—C1	176.26 (16)	C10—C7—C23—O6	−58.77 (17)
C4—C5—C9—C1	−2.7 (2)	C8—C7—C23—O6	58.96 (17)
O1—C5—C9—C8	−0.1 (2)	C12—C11—N1—C14	0.7 (3)
C4—C5—C9—C8	−178.98 (15)	O2—C11—N1—C14	−177.63 (15)
C12—C8—C9—C1	93.78 (18)	C12—C11—N1—C21	−176.51 (17)
C7—C8—C9—C1	−145.05 (15)	O2—C11—N1—C21	5.2 (2)
C12—C8—C9—C5	−90.03 (17)	O3—C14—N1—C11	178.87 (17)
C7—C8—C9—C5	31.15 (19)	N2—C14—N1—C11	−0.2 (2)
C23—C7—C10—O2	−178.60 (12)	O3—C14—N1—C21	−3.9 (3)
C6—C7—C10—O2	−57.87 (15)	N2—C14—N1—C21	177.04 (16)
C8—C7—C10—O2	62.33 (15)	O3—C14—N2—C13	173.81 (18)
C23—C7—C10—C15	−58.56 (16)	N1—C14—N2—C13	−7.1 (3)
C6—C7—C10—C15	62.17 (16)	O3—C14—N2—C22	6.2 (3)
C8—C7—C10—C15	−177.63 (12)	N1—C14—N2—C22	−174.76 (19)
O2—C11—C12—C13	−176.39 (15)	O4—C13—N2—C14	−167.39 (18)
N1—C11—C12—C13	5.6 (2)	C12—C13—N2—C14	13.1 (3)
O2—C11—C12—C8	−1.2 (2)	O4—C13—N2—C22	0.4 (3)
N1—C11—C12—C8	−179.28 (14)	C12—C13—N2—C22	−179.16 (18)
C9—C8—C12—C11	142.93 (15)	C9—C5—O1—C6	−6.3 (2)
C7—C8—C12—C11	22.38 (19)	C4—C5—O1—C6	172.70 (14)
C9—C8—C12—C13	−42.1 (2)	C7—C6—O1—C5	−21.03 (19)
C7—C8—C12—C13	−162.66 (14)	C12—C11—O2—C10	11.6 (2)
C11—C12—C13—O4	168.86 (17)	N1—C11—O2—C10	−170.11 (13)
C8—C12—C13—O4	−6.3 (3)	C15—C10—O2—C11	−168.67 (13)
C11—C12—C13—N2	−11.6 (2)	C7—C10—O2—C11	−42.47 (17)
C8—C12—C13—N2	173.23 (14)	O5—C23—O6—C24	−3.5 (3)
O2—C10—C15—C16	38.0 (2)	C7—C23—O6—C24	176.02 (19)
C7—C10—C15—C16	−84.24 (19)		

### Hydrogen-bond geometry (Å, °)

**Table:**

*Cg*4 is the centroid of the C1–C5/C9 ring.

**Table:**

*D*—H···*A*	*D*—H	H···*A*	*D*···*A*	*D*—H···*A*
C16—H16···O3^i^	0.93	2.44	3.349 (2)	166
C19—H19···Cg4^ii^	0.93	2.84	3.720 (3)	158

Symmetry codes: (i) −*x*+1, −*y*, −*z*; (ii) *x*−1, *y*, *z*.
